# Supplementary material for: Targeted analysis of KRAS and CREBBP mutations uncovers a potential population-specific signature in thai patients with liver fluke-associated cholangiocarcinoma
Source: PLoS One. 2026 May 8;21(5):e0348942. doi: 10.1371/journal.pone.0348942 (PMC13155607; doi:10.1371/journal.pone.0348942)
Supplement: S1 Table — (DOCX) [file pone.0348942.s001.docx]

**S1 Table**. Clinical and genetic data from patients with cholangiocarcinoma and *Opisthorchis viverrini* infection, and from healthy subjects.

| Code | Tumor_size  cm | Metastasis | Stage | TP53 | KRAS 12 | KRAS 13 | CDKN2A | IDH1 | CREBBP | GZMB (rs8192917) | GZMB  (rs2236338) |
| --- | --- | --- | --- | --- | --- | --- | --- | --- | --- | --- | --- |
| CCA1 | 3.7 | 0 | 1 | GG | GG | GA | TT | CC | GG | CC | AA |
| CCA2 |  | 0 |  | GC | GG | GG | TT | CC | GG | CC | AA |
| CCA3 | 1.4 | 0 |  | GG | GG | GG | TT | CC | GG | CC | AA |
| CCA4 | 6.7 | 0 | 3 | GG | GG | GG | TT | CC | GT | CC | AA |
| CCA5 | 3.5 | 1 | 4 | GC | GG | GA | TT | CC | TT | CC | AA |
| CCA6 | 1.4 | 0 | 2 | GC | GG | GA | TT | CC | TT | CC | AA |
| CCA7 | 3 | 0 | 2 | GC | GG | GA | TC | CC | GG | CC | AA |
| CCA8 | 3 | 0 | 2 | CC | GG | GA | TT | CC | GG | CC | AA |
| CCA9 |  | 0 |  | GC | GG | GG | TT | CC | TT | CC | AA |
| CCA10 | 3 | 0 |  | GG | GG | GG | TT | CC | TT | CC | AA |
| CCA11 | 12 | 0 | 3 | GG | GG | GG | TT | CC | GG | CC | AA |
| CCA12 | 7 | 1 | 5 | GC | GG | GA | TT | CC | TT | CC | AA |
| CCA13 | 4.8 | 1 | 5 | GC | GG | GG | TT | CC | GG | CC | AA |
| CCA14 | 2.6 | 1 | 5 | CC | GG | GA | TT | CC | GG | CC | AA |
| CCA15 | 6 | 1 | 5 | CC | GG | GG | TT | CC | TT | CC | AA |
| CCA16 | 13.2 | 1 | 5 | CC | GG | GG | TT | CC | GG | CC | AA |
| CCA17 | 5.9 | 1 | 5 | CC | GG | GA | TT | CC | GT | CC | AA |
| CCA18 | 14.9 | 1 | 5 | CC | GG | GG | TT | CC | GT | CC | AA |
| CCA19 | 6.7 | 1 | 5 | GC | GG | GG | TT | CC | GT | CC | AA |
| CCA20 | 5.9 | 1 | 5 | CC | GG | GG | TT | CC | GT | CC | AA |
| CCA21 | 0.55 | 0 | 3 | GC | GG | GG | TT | CC | GT | CC | AA |
| CCA22 | 1.5 | 1 | 5 | GG | GG | GA | TT | CC | TT | CC | AA |
| CCA23 | 12 | 1 | 5 | CC | GG | GA | TT | CC | GG | CC | AA |
| CCA24 | 5.4 | 1 | 5 | GG | GG | GG | TT | CC | GG | CC | AA |
| CCA25 | 0.03 | 0 | 2 | GC | GG | GA | TT | CC | GT | CC | AA |
| CCA26 | 6.3 | 1 | 5 | GC | GG | GG | TT | CC | GT | CC | AA |
| CCA27 | 7 | 1 | 4 | GC | GG | GG | TT | CC | GG | CC | AA |
| CCA28 | 1.3 | 0 | 2 | GG | GG | GG | TT | CC | GG | CC | AA |
| CCA29 | 13.2 | 1 | 5 | GC | GG | GG | TT | CC | GG | CC | AA |
| CCA30 | 6.9 | 1 | 5 | CC | GG | GG | TT | CC | GT | CC | AA |
| CCA31 | 3.7 | 1 | 5 | GC | GG | GA | TT | CC | GT | CC | AA |
| CCA32 | 7.5 | 1 | 5 | GC | GG | GG | TT | CC | GT | CC | AA |
| CCA33 | 3.6 | 1 | 5 | GC | GG | GG | TT | CC | GG | CC | AA |
| CCA34 | 11.6 | 1 | 5 | GG | GG | GG | TT | CC | GT | CC | AA |
| CCA35 | 2.3 | 1 | 5 | GG | GG | GG | TT | CC | GG | CC | AA |
| CCA36 |  |  |  | GC | GG | GG | TT | CC | GG | CT | AA |
| CCA37 |  |  |  | CC | GG | GG | TT | CC | GG | CC | AA |
| CCA38 |  |  |  | GC | GG | GG | TT | CC | GG | CC | AA |
| CCA39 |  |  |  | GC | GG | GG | TT | CC | TT | CC | AA |
| CCA40 |  |  |  | GG | GG | GG | TT | CC | GG | CC | AA |
| CCA41 |  |  |  | GC | GG | GA | TT | CC | GT | CC | AA |
| CCA42 |  |  |  | GC | GG | GG | TT | CC | GG | CC | AA |
| CCA43 |  |  |  | CC | GG | GA | TT | CC | GG | CC | AA |
| CCA44 |  |  |  | GG | GG | GG | TT | CC | GG | CC | AA |
| CCA45 |  |  |  | GC | GG | GA | TT | CC | GG | CC | AA |
| CCA46 |  |  |  | GC | GG | GG | TT | CC | GG | CC | AA |
| CCA47 |  |  |  | GC | GG | GG | TT | CC | GG | CC | AA |
| CCA48 |  |  |  | GG | GG | GG | TT | CC | GT | CC | AA |
| CCA49 |  |  |  | GC | GG | GG | TT | CC | GT | CC | AA |
| CCA50 |  |  |  | CC | GG | GG | TT | CC | GT | CC | AA |
| CCA51 |  |  |  | GC | GG | GG | TT | CC | GG | CC | AA |
| CCA52 |  |  |  | GC | GG | GG | TT | CC | GT | CC | AA |
| CCA53 |  |  |  | CC | GG | GG | TT | CC | GT | CC | AA |
| CCA54 |  |  |  | CC | GG | GG | TT | CC | GT | CC | AA |
| CCA55 |  |  |  | GG | GG | GG | TT | CC | GT | CC | AA |
| CCA56 |  |  |  | GG | GG | GG | TT | CC | TT | CC | AA |
| CCA57 |  |  |  | GC | GG | GA | TT | CC | GT | CC | AA |
| CCA58 |  |  |  | GC | GG | GA | TT | CC | GT | CC | AA |
| CCA59 |  |  |  | GG | GG | GG | TT | CC | GT | CC | AA |
| CCA60 |  |  |  | GC | GG | AA | TT | CC | GG | CT | AA |
| CCA61 |  |  |  | GG | GG | GA | TT | CC | GT | CC | AA |
| CCA62 |  |  |  | CC | GG | GA | TT | CC | GT | CT | AA |
| CCA63 |  |  |  | GG | GG | GG | TT | CC | GG | CC | AA |
| CCA64 |  |  |  | GC | GG | GG | TT | CC | GT | CC | AA |
| CCA65 |  |  |  | GC | GG | GG | TT | CC | GG | CC | AA |
| CCA66 |  |  |  | CC | GG | GG | TT | CC | GT | CC | AA |
| CCA67 |  |  |  | GG | GG | GG | TT | CC | GG | CC | AA |
| CCA68 |  |  |  | CC | GG | GA | TT | CC | GT | CC | AA |
| CCA69 |  |  |  | GC | GG | AA | TT | CC | GT | CC | AA |
| CCA70 |  |  |  | CC | GG | GA | TT | CC | GG | CC | AA |
| CCA71 |  |  |  | GG | GG | GG | TT | CC | GG | CC | AA |
| CCA72 |  |  |  | GG | GG | GG | TT | CC | GG | CC | AA |
| CCA73 |  |  |  | GC | GG | GG | TT | CC | GG | CT | AA |
| CCA74 |  |  |  | GC | GG | GG | TT | CC | GG | CC | AA |
| CCA75 |  |  |  | GC | GG | GG | TT | CC | TT | CC | AA |
| CCA76 |  |  |  | CC | GG | GG | TT | CC | GG | CC | AA |
| CCA77 |  |  |  | GG | GG | GG | TT | CC | GT | CC | AA |
| CCA78 |  |  |  | GC | GG | GG | TT | CC | GG | CC | AA |
| CCA79 |  |  |  | GC | GG | GG | TT | CC | GG | CC | AA |
| CCA80 |  |  |  | GC | GG | GG | TT | CC | GG | CC | AA |
| CCA81 |  |  |  | GC | GG | GG | TT | CC | GG | CC | AA |
| CCA82 |  |  |  | GG | GG | GG | TT | CC | GG | CC | AA |
| CCA83 |  |  |  | GC | GG | GG | TT | CC | GG | CC | AA |
| CCA84 |  |  |  | GC | GG | GG | TT | CC | GG | CC | AA |
| CCA85 |  |  |  | CC | GG | GG | TT | CC | GT | CC | AA |
| CCA86 |  |  |  | GC | GG | GG | TT | CC | GG | CC | AA |
| CCA87 |  |  |  | GG | GG | GG | TT | CC | GT | CC | AA |
| CCA88 |  |  |  | GC | GG | GG | TT | CC | GT | CT | AA |
| CCA89 |  |  |  | GC | GG | GG | TT | CC | GT | CC | AA |
| CCA90 |  |  |  | CC | GG | GG | TT | CC | GT | CC | AA |
| CCA91 |  |  |  | CC | GG | GG | TT | CC | GG | CC | AA |
| CCA92 |  |  |  | GC | GG | GG | TT | CC | GG | CC | AA |
| CCA93 |  |  |  | CC | GG | GG | TT | CC | GT | CC | AA |
| CCA94 |  |  |  | GC | GG | GG | TT | CC | GT | CC | AA |
| CCA95 |  |  |  | GC | GG | GG | TT | CC | GG | CC | AA |
| CCA96 |  |  |  | GC | GG | GG | TT | CC | GG | CC | AA |
| CCA97 |  |  |  | GC | GG | GG | TT | CC | GG | CC | AA |
| CCA98 |  |  |  | CC | GG | GG | TT | CC | GT | CC | AA |
| CCA99 |  |  |  | GC | GG | GG | TT | CC | GT | CC | AA |
| CCA100 |  |  |  | GC | GG | GG | TT | CC | GT | CC | AA |
| CCA101 |  |  |  | GC | GG | GG | TT | CC | GG | CC | AA |
| CCA102 |  |  |  | GG | GG | GG | TT | CC | GT | CC | AA |
| CCA103 |  |  |  | GC | GG | GG | TT | CC | GG | CC | AA |
| CCA104 |  |  |  | GG | GG | GG | TT | CC | GT | CC | AA |
| CCA105 |  |  |  | GC | GG | GG | TT | CC | GT | CC | AA |
| CCA106 |  |  |  | GG | GG | GG | TT | CC | GG | CC | AA |
| CCA107 |  |  |  | GC | GG | GG | TT | CC | GT | CC | AA |
| CCA108 |  |  |  | GG | GG | GG | TT | CC | GT | CC | AA |
| CCA109 |  |  |  | GC | GG | GG | TT | CC | GG | CC | AA |
| CCA110 |  |  |  | GC | GG | GG | TT | CC | GG | CC | AA |

**Supporting Information: Patients with *Opisthorchis viverrini* infection**

| **Code** | **TP53** | **KRAS 12** | **KRAS 13** | **CDKN2A** | **IDH1** | **CREBBP** | **GZMB (rs8192917)** | **GZMB (rs2236338)** |
| --- | --- | --- | --- | --- | --- | --- | --- | --- |
| OV001 | CC | GG | GG | TT | CC | GT | CC | GG |
| OV002 | GC | GG | GG | TT | CC | GT | CC | GG |
| OV003 | CC | GG | GG | TT | CC | GT | CC | GG |
| OV004 | GC | GG | GG | TT | CC | GG | CC | GG |
| OV005 | GC | GG | GG | TT | CC | GT | CC | GG |
| OV006 | GC | GG | GG | TT | CC | GT | CC | GG |
| OV007 | CC | GG | GG | TT | CC | GG | CC | GG |
| OV008 | CC | GG | GG | TT | CC | GT | CC | GG |
| OV009 | GC | GG | GG | TT | CC | GT | CC | GG |
| OV010 | CC | GG | GG | TT | CC | GT | CC | GG |
| OV011 | CC | GG | GG | TT | CC | GT | CC | GG |
| OV012 | GC | GG | GG | TT | CC | GT | CC | GG |
| OV013 | GG | GG | GG | TT | CC | GG | CC | GG |
| OV014 | GC | GG | GG | TT | CC | GT | CC | GG |
| OV015 | CC | GG | GG | TT | CC | GT | CC | GG |
| OV016 | GC | GG | GG | TT | CC | GG | CC | GG |
| OV017 | GC | GG | GG | TT | CC | GT | CC | GG |
| OV018 | CC | GG | GG | TT | CC | GG | CC | GG |
| OV019 | GC | GG | GG | TT | CC | GT | CC | GG |
| OV020 | GC | GG | GG | TT | CC | GG | CC | GG |
| OV021 | GC | GG | GG | TT | CC | GT | CC | GG |
| OV022 | GC | GG | GG | TT | CC | GT | CC | GG |
| OV023 | CC | GG | GG | TT | CC | GG | CC | GG |
| OV024 | GG | GG | GG | TT | CC | GT | CC | GG |
| OV025 | GC | GG | GG | TT | CC | GG | CC | GG |
| OV026 | GG | GG | GG | TT | CC | GG | CC | GG |
| OV027 | CC | GG | GG | TT | CC | GG | CC | GG |
| OV028 | GC | GG | GG | TT | CC | GG | CC | GG |
| OV029 | GC | GG | GG | TT | CC | GG | CC | GG |
| OV030 | GC | GG | GG | TT | CC | GG | CC | GG |
| OV031 | GG | GG | GG | TT | CC | GG | CC | GG |
| OV032 | GC | GG | GG | TT | CC | GG | CC | GG |
| OV033 | CC | GG | GG | TT | CC | GG | CC | GG |
| OV034 | CC | GG | GG | TT | CC | GG | CC | GG |
| OV035 | GC | GG | GG | TT | CC | GG | CC | GG |
| OV036 | GC | GG | GG | TT | CC | GT | CC | GG |
| OV037 | GC | GG | GG | TT | CC | GG | CC | GG |
| OV038 | GG | GG | GG | TT | CC | GG | CC | GG |
| OV039 | GG | GG | GG | TT | CC | GG | CC | GG |
| OV040 | GG | GG | GG | TT | CC | GG | CC | GG |
| OV041 | GC | GG | GG | TT | CC | GG | CC | GG |
| OV042 | CC | GG | GG | TT | CC | GG | CC | GG |
| OV043 | GC | GG | GG | TT | CC | GG | CC | GG |
| OV044 | CC | GG | GG | TT | CC | GG | CC | GG |
| OV045 | GC | GG | GG | TT | CC | GG | CC | GG |
| OV046 | CC | GG | GG | TT | CC | GG | CC | GG |
| OV047 | CC | GG | GG | TT | CC | GG | CC | GG |
| OV048 | CC | GG | GG | TT | CC | GG | CC | GG |
| OV049 | GG | GG | GG | TT | CC | GG | CC | GG |
| OV050 | GG | GG | GG | TT | CC | GG | CC | GG |
| OV051 | CC | GG | GG | TT | CC | GG | CC | GG |
| OV052 | GC | GG | GG | TT | CC | GG | CC | GG |
| OV053 | GG | GG | GG | TT | CC | GG | CC | GG |
| OV054 | GG | GG | GG | TT | CC | GG | CC | GG |
| OV055 | GG | GG | GG | TT | CC | GG | CC | GG |
| OV056 | GC | GG | GG | TT | CC | GG | CC | GG |
| OV057 | GC | GG | GG | TT | CC | GG | CC | GG |
| OV058 | GG | GG | GG | TT | CC | GG | CC | GG |
| OV059 | CC | GG | GG | TT | CC | GG | CC | GG |
| OV060 | GC | GG | GG | TT | CC | GG | CC | GG |

**Supporting Information: Healthy subjects**

| Code | TP53 | KRAS 12 | KRAS 13 | CDKN2A | IDH1 | CREBBP | GZMB (rs8192917) | GZMB (rs2236338) |
| --- | --- | --- | --- | --- | --- | --- | --- | --- |
| H001 | GG | GG | GG | TT | CC | GG | CC | GG |
| H002 | GC | GG | GG | TT | CC | GG | CC | GG |
| H003 | GG | GG | GG | TT | CC | GG | CC | GG |
| H004 | CC | GG | GG | TT | CC | GG | CC | GG |
| H005 | CC | GG | GG | TT | CC | GG | CC | GG |
| H006 | GG | GG | GG | TT | CC | GG | CC | GG |
| H007 | CC | GG | GG | TT | CC | GT | CC | GG |
| H008 | GG | GG | GG | TT | CC | GT | CC | GG |
| H009 | GC | GG | GG | TT | CC | GG | CC | GG |
| H010 | GG | GG | GG | TT | CC | GT | CC | GG |
| H011 | GC | GG | GG | TT | CC | GG | CC | GG |
| H012 | CC | GG | GG | TT | CC | GT | CC | GG |
| H013 | GG | GG | GG | TT | CC | GT | CC | GG |
| H014 | CC | GG | GG | TT | CC | GT | CC | GG |
| H015 | CC | GG | AA | TT | CC | GT | CC | GG |
| H016 | GC | GG | GG | TT | CC | GT | CC | GG |
| H017 | CC | GG | GG | TT | CC | GG | CC | GG |
| H018 | GG | GG | GG | TT | CC | GT | CC | GG |
| H019 | CC | GG | GG | TT | CC | GT | CC | GG |
| H020 | GG | GG | GG | TT | CC | GT | CC | GG |
| H021 | CC | GG | GG | TT | CC | GT | CC | GG |
| H022 | GC | GG | GG | TT | CC | GG | CC | GG |
| H023 | GC | GG | GG | TT | CC | TT | CC | GG |
| H024 | GG | GG | GG | TT | CC | GT | CC | GG |
| H025 | GC | GG | GG | TT | CC | GG | CC | GG |
| H026 | CC | GG | GG | TT | CC | GT | TT | GG |
| H027 | CC | GG | GG | TT | CC | GT | CC | GG |
| H028 | GC | GG | GG | TT | CC | GT | CC | GG |
| H029 | GC | GG | GG | TT | CC | GG | CC | GG |
| H030 | GC | GG | GG | TT | CC | GG | CC | GG |
| H031 | GC | GG | GG | TT | CC | GG | CC | GG |
| H032 | GC | GG | GG | TT | CC | GG | CC | GG |
| H033 | CC | GG | GG | TT | CC | GG | CC | GG |
| H034 | GG | GG | GG | TT | CC | GG | CC | GG |
| H035 | GC | GG | GG | TT | CC | TT | CC | GG |
| H036 | GC | GG | GG | TT | CC | GG | CC | GG |
| H037 | CC | GG | GG | TT | CC | GG | CC | GG |
| H038 | GG | GG | GG | TT | CC | GG | CC | GG |
| H039 | CC | GG | AA | TT | CC | GG | CC | GG |
| H040 | GG | GG | GG | TT | CC | GG | CC | GG |
| H041 | GC | GG | GG | TT | CC | GG | CC | GG |
| H042 | CC | GG | GA | TT | CC | GG | CC | GG |
| H043 | CC | GG | GG | TT | CC | GG | CC | GG |
| H044 | GC | GG | GG | TT | CC | GG | CC | GG |
| H045 | GG | GG | GG | TT | CC | GG | CC | GG |
| H046 | CC | GG | GG | TT | CC | GG | CC | GG |
| H047 | GC | GG | GG | TT | CC | GG | CC | GG |
| H048 | GG | GG | GG | TT | CC | GG | CC | GG |
| H049 | GC | GG | AA | TT | CC | GG | CC | GG |
| H050 | GG | GG | GA | TT | CC | GG | CC | GG |
| H051 | GC | GG | GA | TT | CC | GG | CC | GG |
| H052 | GG | GG | GA | TT | CC | GG | CC | GG |
| H053 | GC | GG | GA | TT | CC | GG | CC | GG |
| H054 | GC | GG | GA | TT | CC | GG | CC | GG |
| H055 | GC | GG |  | TT | CC | GG | CC | GG |
| H056 | GG | GG | GA | TT | CC | GG | CC | GG |
| H057 | CC | GG | GA | TT | CC | GG | CC | GG |
| H058 | GC | GG |  | TT | CC | GG | CC | GG |
| H059 | GG | GG | GA | TT | CC | GG | CC | GG |
| H060 | CC | GG | GA | TT | CC | GG | CC | GG |
| H061 | CC | GG | AA | TT | CC | GG | CC | GG |
| H062 | GG | GG | No product | TT | CC | GG | CC | GG |
| H063 | GC | GG | GA | TT | CC | GG | CC | GG |
| H064 | CC | GG | GA | TT | CC | GT | CC | GG |
| H065 | GC | GG | GA | TT | CC | GT | CC | GG |
| H066 | GC | GG | GA | TT | CC | GG | CC | GG |
| H067 | GC | GG | GA | TT | CC | GT | CC | GG |
| H068 | GC | GG | GA | TT | CC | GT | CC | GG |
| H069 | GC | GG | GA | TT | CC | GT | CC | GG |
| H070 | GG | GG | GA | TT | CC | GT | CC | GG |
| H071 | CC | GG | GA | TT | CC | GT | CC | GG |
| H072 | CC | GG | GG | TT | CC | GG | CC | GG |
| H073 | CC | GG | GG | TT | CC | GG | CC | GG |
| H074 | CC | GG | GG | TT | CC | GG | CC | GG |
| H075 | GG | GG | GG | TT | CC | GG | CC | GG |
| H076 | GC | GG | GG | TT | CC | GG | CC | GG |
| H077 | CC | GG | AA | TT | CC | GT | CC | GG |
| H078 | CC | GG | GA | TT | CC | GT | CC | GG |
| H079 | GG | GG | GA | TT | CC | GT | CC | GG |
| H080 | CC | GG | AA | TT | CC | GT | CC | GG |
| H081 | GC | GG | GG | TT | CC | GT | CC | GG |
| H082 | GG | GG | GG | TT | CC | GG | CC | GG |
| H083 | GC | GG | GG | TT | CC | GG | CC | GG |
| H084 | GC | GG | GG | TT | CC | GG | CC | GG |
| H085 | GC | GG | GG | TT | CC | GT | CC | GG |
| H086 | GC | GG | GG | TT | CC | GT | CC | GG |
| H087 | GC | GG | GG | TT | CC | GT | CC | GG |
| H088 | GC | GG | GG | TT | CC | GT | CC | GG |
| H089 | GG | GG | GG | TT | CC | GT | CC | GG |
| H090 | GG | GG | GG | TT | CC | GG | CC | GG |
| H091 | CC | GG | GG | TT | CC | GG | CC | GG |
| H092 | GC | GG | GG | TT | CC | GT | CC | GG |
| H093 | GG | GG | GG | TT | CC | GT | CC | GG |
| H094 | GG | GG | GG | TT | CC | GG | CC | GG |
| H095 | GC | GG | GG | TT | CC | TT | CC | GG |
| H096 | GG | GG | GG | TT | CC | GG | CC | GG |
| H097 | CC | GG | GG | TT | CC | GG | CC | GG |
| H098 | GC | GG | GG | TT | CC | GT | CC | GG |
| H099 | CC | GG | GG | TT | CC | GT | CC | GG |
| H100 | GG | GG | GG | TT | CC | GT | CC | GG |
| H101 | GC | GG | GG | TT | CC | GT | CC | GG |
| H102 | GC | GG | GG | TT | CC | GG | CC | GG |
| H103 | GC | GG | GG | TT | CC | GT | CC | GG |
| H104 | GC | GG | GG | TT | CC | GG | CC | GG |
| H105 | GG | GG | GG | TT | CC | GT | CC | GG |
| H106 | GG | GG | GG | TT | CC | GG | CC | GG |
| H107 | GG | GG | GG | TT | CC | GG | CC | GG |
| H108 | GC | GG | GG | TT | CC | GG | CC | GG |
| H109 | CC | GG | GG | TT | CC | GG | CC | GG |
| H110 | CC | GG | GG | TT | CC | GG | CC | GG |
| H111 | GC | GG | GG | TT | CC | GG | CC | GG |
| H112 | GG | GG | GG | TT | CC | GG | CC | GG |
| H113 | GC | GG | GG | TT | CC | GG | CC | GG |
| H114 | CC | GG | GG | TT | CC | GG | CC | GG |
| H115 | GC | GG | GG | TT | CC | GG | CC | GG |
| H116 | CC | GG | GG | TT | CC | GG | CC | GG |
| H117 | GG | GG | GG | TT | CC | GG | CC | GG |
| H118 | GC | GG | GG | TT | CC | GG | CC | GG |
| H119 | GC | GG | GG | TT | CC | GG | CC | GG |
| H120 | GC | GG | GG | TT | CC | GG | CC | GG |
| H121 | GG | GG | GG | TT | CC | GG | CC | GG |
| H122 | GC | GG | GG | TT | CC | GG | CC | GG |
| H123 | GG | GG | GG | TT | CC | GG | CC | GG |
| H124 | GC | GG | GG | TT | CC | GG | CC | GG |
| H125 | CC | GG | GG | TT | CC | GG | CC | GG |
| H126 | GG | GG | GG | TT | CC | GG | CC | GG |
| H127 | GC | GG | GG | TT | CC | GG | CC | GG |
| H128 | CC | GG | GG | TT | CC | GG | CC | GG |
| H129 | GC | GG | GG | TT | CC | GG | CC | GG |
| H130 | GC | GG | GG | TT | CC | GG | CC | GG |
| H131 | GG | GG | GG | TT | CC | GG | CC | GG |
| H132 | GG | GG | GG | TT | CC | GG | CC | GG |
| H133 | GG | GG | GG | TT | CC | GG | CC | GG |
| H134 | GG | GG | GG | TT | CC | GG | CC | GG |
| H135 | GG | GG | GG | TT | CC | GG | CC | GG |
| H136 | CC | GG | GG | TT | CC | GG | CC | GG |
| H137 | GC | GG | GG | TT | CC | GG | CC | GG |
| H138 | CC | GG | GG | TT | CC | GG | CC | GG |
| H139 | GG | GG | GG | TT | CC | GG | CC | GG |
| H140 | GC | GG | GG | TT | CC | GG | CC | GG |
| H141 | GC | GG | GG | TT | CC | GG | CC | GG |
| H142 | GC | GG | GG | TT | CC | GG | CC | GG |
| H143 | GG | GG | GG | TT | CC | GG | CC | GG |
| H144 | GC | GG | GG | TT | CC | GG | CC | GG |
| H145 | GG | GG | GG | TT | CC | GG | CC | GG |
| H146 | CC | GG | GG | TT | CC | GG | CC | GG |
| H147 | CC | GG | GG | TT | CC | GG | CC | GG |
| H148 | GG | GG | GG | TT | CC | GG | CC | GG |
| H149 | GC | GG | GG | TT | CC | GT | CC | GG |
| H150 | GG | GG | GG | TT | CC | GT | CC | GG |
| H151 | GC | GG | GG | TT | CC | GG | CC | GG |
| H152 | GG | GG | GG | TT | CC | GG | CC | GG |
| H153 | GC | GG | GG | TT | CC | GG | CC | GG |
| H154 | CC | GG | GG | TT | CC | GT | CC | GG |
| H155 | GC | GG | GG | TT | CC | GG | CC | GG |
| H156 | CC | GG | GG | TT | CC | GT | CC | GG |
