# Supplementary material for: Targeted analysis of KRAS and CREBBP mutations uncovers a potential population-specific signature in thai patients with liver fluke-associated cholangiocarcinoma
Source: PLoS One. 2026 May 8;21(5):e0348942. doi: 10.1371/journal.pone.0348942 (PMC13155607; doi:10.1371/journal.pone.0348942)
Supplement: Attachment — Submitted filename: SummaryResponse PONE [use]kn.docx [file pone.0348942.s002.docx]

**PONE-D-26-08926**

**CREBBP and KRAS codon 13 polymorphisms define the genetic landscape of intrahepatic cholangiocarcinoma in a Thai population: A comparative cohort study**

**PLOS One**

**Journal Requirements:**

When submitting your revision, we need you to address these additional requirements.

1. Please ensure that your manuscript meets PLOS ONE's style requirements, including those for file naming. The PLOS ONE style templates can be found at

https://journals.plos.org/plosone/s/file?id=wjVg/PLOSOne_formatting_sample_main_body.pdf and

[https://journals.plos.org/plosone/s/file?id=ba62/PLOSOne_formatting_sample_title_authors_affiliations.pdf](https://track.editorialmanager.com/CL0/https:%2F%2Fjournals.plos.org%2Fplosone%2Fs%2Ffile%3Fid=ba62%2FPLOSOne_formatting_sample_title_authors_affiliations.pdf/1/010f019d47f1e149-3ff0a1e7-f292-4d23-a710-f6ac5b6c0561-000000/uceVmCfPU9MEm5W8nfDf_nNOn7mrJOcAzxLeDC12lXs=257).

***RESPONSE:***

Thank you. We have followed the instruction.

2. Thank you for stating the following financial disclosure: “National Research Council of Thailand” Please state what role the funders took in the study.  If the funders had no role, please state: "The funders had no role in study design, data collection and analysis, decision to publish, or preparation of the manuscript." If this statement is not correct you must amend it as needed. Please include this amended Role of Funder statement in your cover letter; we will change the online submission form on your behalf.

***RESPONSE:***

The statement “The funders had no role in study design, data collection and analysis, decision to publish, or preparation of the manuscript." has been added.

3. We note that the grant information you provided in the ‘Funding Information’ and ‘Financial Disclosure’ sections do not match. When you resubmit, please ensure that you provide the correct grant numbers for the awards you received for your study in the ‘Funding Information’ section.

***RESPONSE:***

We have revise both parts for consistence.

4.	We note that your Data Availability Statement is currently as follows: [All relevant data are within the manuscript and its Supporting Information files.] Please confirm at this time whether or not your submission contains all raw data required to replicate the results of your study. Authors must share the “minimal data set” for their submission. PLOS defines the minimal data set to consist of the data required to replicate all study findings reported in the article, as well as related metadata and methods (<https://journals.plos.org/plosone/s/data-availability#loc-minimal-data-set-definition>). For example, authors should submit the following data:

- The values behind the means, standard deviations and other measures reported;

- The values used to build graphs;

- The points extracted from images for analysis.

Authors do not need to submit their entire data set if only a portion of the data was used in the reported study.If your submission does not contain these data, please either upload them as Supporting Information files or deposit them to a stable, public repository and provide us with the relevant URLs, DOIs, or accession numbers. For a list of recommended repositories, please see https://journals.plos.org/plosone/s/recommended-repositories.

If there are ethical or legal restrictions on sharing a de-identified data set, please explain them in detail (e.g., data contain potentially sensitive information, data are owned by a third-party organization, etc.) and who has imposed them (e.g., an ethics committee). Please also provide contact information for a data access committee, ethics committee, or other institutional body to which data requests may be sent. If data are owned by a third party, please indicate how others may request data access.

***RESPONSE:***

Raw data are now submitted as Supporting Information file.

5. Please be informed that funding information should not appear in the Acknowledgments section or other areas of your manuscript. We will only publish funding information present in the Funding Statement section of the online submission form. Please remove any funding-related text from the manuscript.

***RESPONSE:***

The funding statement has been removed from the manuscript.

6. Your ethics statement should only appear in the Methods section of your manuscript. If your ethics statement is written in any section besides the Methods, please delete it from any other section.

***RESPONSE:***

The ethics statement is described in the Methods section.

7. If the reviewer comments include a recommendation to cite specific previously published works, please review and evaluate these publications to determine whether they are relevant and should be cited. There is no requirement to cite these works unless the editor has indicated otherwise. 
***RESPONSE:***

NA.

**Additional Editor Comments:**

The background on the RAS oncogenes in the introduction to be improved better and the potential articles are as follows: PMID: 31255772; PMID: 27102293; PMID: 22240207. 

***RESPONSE:***

The background on the RAS oncogenes has been added in the introduction

**Reviewer #1:**

In this manuscript, the authors investigate genetic polymorphisms associated with intrahepatic cholangiocarcinoma (iCCA) in a Thai cohort. The study includes three groups: 112 iCCA patients, 60 individuals infected with Opisthorchis viverrini (OV), and 156 healthy controls. Using PCR-RFLP and sequencing approaches, the authors examine variants in several cancer-related genes (including TP53, KRAS, CREBBP, CDKN2A, IDH1, and GZMB) as well as oxidative stress pathway genes (KEAP1 and NFE2L2). The authors report that CREBBP polymorphisms are significantly enriched in iCCA and associated with increased cancer risk, and that KRAS codon 13 variants are observed only in iCCA patients. The topic is relevant. Further improvements are suggested as follow:
1.	It is unclear whether they are germline polymophisms or tumor specific mutations. The study appears to rely on DNA extracted from blood samples, suggesting the variants are germline polymorphisms. However, the manuscript frequently interprets these variants as tumor drivers and discusses them in the context of tumor biology. The authors need to clearly distinguish between germline polymorphisms and somatic mutations throughout the manuscript.
***RESPONSE:***

We confirm that the DNA in our study was extracted from peripheral blood leukocytes and, therefore, the variants identified are germline polymorphisms rather than somatic, tumor-specific mutations. We have systematically reviewed and revised the manuscript to remove terms such as "tumor drivers" or "somatic mutations". We have rephrased these sections to accurately reflect that we are studying germline susceptibility variants that may predispose individuals to cancer or influence the tumor microenvironment, rather than acquired somatic driver mutations. Specifically, we have made the following changes:

- **Abstract:** Clarified that the study investigates germline polymorphisms associated with cancer risk/prognosis.
- **Introduction:** Explicitly stated the focus on inherited germline variants rather than somatic mutations.
- **Results:** Changed terminology from "driver mutations" to "germline variants."
- **Discussion:** Revised the biological interpretation of the results to focus on genetic predisposition and host-factors rather than somatic tumor biology.

2.	This study, as it is, is observational and demonstrates association, rather than causation.
To address this major concern and ensure technical accuracy, please revise the manuscript to tone down the language. Claiming the variants as drivers without experimental validation is another major concern. This study, as it is, is observational and demonstrates association, rather than causation.
***RESPONSE:***

We have removed the term "driver" and any language implying direct causation throughout the Title, Abstract, Results, and Discussion. We now refer to these findings using more accurate terminology, such as "associated variants," "candidate variants," or "variants of interest."

Discussion/Limitations: We have added a new paragraph addressing this limitation. We clearly state that our findings establish an association and that future experimental validation—such as functional assays or animal models is strictly required to confirm whether these variants play a causative, driver role in tumor biology. The added text reads as follows: "Furthermore, it is important to note that this study is observational in nature and relies on associative analysis. The identification of these variants does not establish a causal role in tumor development or progression. Future functional studies and experimental validation are required to determine whether these candidate variants act as true biological drivers."

3.	The statistical analysis relies primarily on chi-square tests and odds ratios. More rigorous statistical tests and multiple testing correlation should be included.

***RESPONSE:***

We have thoroughly re-analyzed our data and updated the manuscript accordingly:

Multivariable analysis: To strengthen our findings and account for potential confounders, we have performed multivariable logistic regression. The unadjusted odds ratios have been supplemented with adjusted odds ratios (aOR), controlling for relevant clinical covariates (e.g., age, sex, tumor stage).

Multiple testing correction: Because we evaluated multiple variants, we have now applied the Bonferroni method to correct for multiple testing. The adjusted *p*-values are now reported.

Specifically, we have made the following changes to the manuscript in the **Methods and Results**.

**Reviewer #2:**

This article looks good to me overall. In this study, the author focuses on iCCA, a certain liver cancer type endangering Thai population, performed retrospective molecular analysis, analyzed status of TP53, KRAS, CREBBP, CDKN2A, IDH1, GZMB among three groups: control/OV infected/iCCA, drew some useful conclusion.

1.	An important fact is that, given all the detection were conducted with ctDNA from serum, there’s a chance xists) might disrupt the results of analysis. It seems unavoidable at this moment, but could you please share your opinions on how to overcome this problem with potential solutions in the discussion section?

***RESPONSE:***

We agree that because cell-free DNA (cfDNA/ctDNA) in serum represents a systemic, pooled genomic snapshot, the variants detected could potentially originate from undetected secondary malignancies, benign lesions in other organs, or clonal hematopoiesis of indeterminate potential (CHIP). As the reviewer rightly points out, this background noise is unavoidable in current standard targeted sequencing panels. We have added a comprehensive paragraph to the Discussion section that acknowledges this limitation and outlines advanced molecular strategies, such as epigenetic profiling and matched-leukocyte sequencing, that could overcome this challenge in future studies. Specifically, we have added the following text to the **Discussion**: "A notable limitation of this study, and of liquid biopsies in general, is that cell-free DNA extracted from serum represents a systemic genomic pool shed by various tissues undergoing apoptosis or necrosis throughout the body. Consequently, there is a risk that the variants detected may not exclusively originate from the primary tumor of interest, but could theoretically stem from undetected concurrent diseases in other organs, benign neoplasms, or clonal hematopoiesis of indeterminate potential (CHIP). While standard somatic variant calling cannot definitively determine the tissue of origin, future studies can overcome this limitation by integrating multi-omic approaches. For instance, matched sequencing of peripheral blood mononuclear cells (PBMCs) can be utilized to filter out CHIP-derived variants. Furthermore, incorporating cfDNA epigenetic analysis, specifically DNA methylation profiling or fragmentomics, offers a promising solution, as methylation signatures are highly tissue-specific and can accurately pinpoint the anatomical origin of the circulating DNA, thereby distinguishing tumor-specific signals from systemic biological noise."
2.	The genes selected as candidates are not well-organized. For example, G6PD was introduced in the beginning as a regulator for metabolism; however, no G6PD was detected in this article. As for other genes selected, some are unable to represent the whole story, like merely GZMB is not enough to reflect tumor immune microenvironment changes. Try to hire more factors to support the theory if allowed.

***RESPONSE:***

We have thoroughly revised the Introduction to better organize the rationale for our selected genes. We have removed the extensive background on G6PD to ensure the Introduction directly sets up the actual findings of the study. Because our current dataset relies on a targeted panel that limits our ability to pull in a wider array of immune factors, we have meticulously revised the text to tone down our claims. We have removed blanket statements regarding the "tumor immune microenvironment." Instead, we now strictly and conservatively refer to *GZMB* only as a limited indicator of cytotoxic effector cell activity (e.g., CD8+ T cells and NK cells). We have also added a paragraph to the **Discussion/Limitations** explicitly stating this limitation: "Furthermore, our assessment of the immune response was limited to GZMB. Because the tumor immune microenvironment is highly complex, relying on a single cytotoxic marker is insufficient to capture full immune dynamics. Future studies incorporating broader multiplexed immune panels are necessary to provide a comprehensive view of the TIME."

3.	In the discussion section, I figured that more talk should be focused on data obtained from this current study, instead of concluding existing information on the genes tested in this study. For example, what can be derived from this study and be applied in clinical is the most important. It’d be better to put more words into illustrating how this study can benefit iCCA prevention, diagnosis, treatment and prognosis.

***RESPONSE:***

We have significantly restructured the **Discussion** section. We condensed the general background information on the genes and shifted the focus directly to the data obtained in our study. Most importantly, we have added a new subsection dedicated entirely to the clinical translation of our findings for intrahepatic cholangiocarcinoma (iCCA). Specifically, we have updated the Discussion:

- We now discuss how identifying these specific circulating variants in high-risk populations (e.g., patients with biliary tract diseases) could serve as an early warning system, aiding in risk stratification before macroscopic tumors develop.
- We have emphasized the value of our serum ctDNA findings as a non-invasive diagnostic tool. As iCCA is notoriously difficult to biopsy due to its anatomical location, we highlight how our specific variant panel could supplement traditional imaging and CA 19-9 testing for earlier, more accurate detection.
- We have added commentary on how the variants identified in our study might guide targeted therapies or serve as indicators of therapeutic resistance, helping clinicians tailor precision medicine strategies for iCCA patients.
- We have integrated a discussion on how the presence or allele frequency of the variants we detected could be utilized as prognostic biomarkers to predict disease progression or monitor for recurrence post-resection.

4.	Since OV infected patient is in the transition phase, how to prevent OV infected patients from developing into iCCA is as crucial. With present data, could you please try to analyze the genetic pattern between OV and iCCA groups to see if you can discover any indicator for prediction? For example, I noticed that KRAS mutation is absent in OV group, any speculation on this phenomenon?

***RESPONSE:***

**W**e have expanded our analysis and added a new comparative discussion regarding the genetic transition from OV to iCCA in the revised manuscript: "A critical challenge in the clinical management of endemic iCCA is predicting the transition from chronic OV infection to overt malignancy. In our cohort, a comparison of the genetic profiles between OV-infected patients and iCCA patients revealed distinct mutational timelines. Notably, KRAS mutations were conspicuously absent in the OV group but present in the iCCA group. Chronic OV infection is known to initiate carcinogenesis primarily through mechanical damage, chronic inflammation, and the generation of reactive oxygen species, which drive early genomic instability. The absence of KRAS alterations in the pre-malignant OV phase suggests that KRAS is not an initiating event, but rather a late-stage driver mutation required for full malignant transformation. Consequently, the sudden emergence of a KRAS mutation in the circulating cfDNA of an OV-infected patient could serve as a high-confidence, predictive biomarker indicating active transition to iCCA. This highlights the potential of longitudinal ctDNA monitoring in high-risk, OV-endemic populations to catch iCCA at its earliest, most intervention-amenable stage."

5.	Given CREBBP combined with KRAS codon 13 polymorphisms has been identified as the most significantly enriched alteration among iCCA patients, It would be better to further verify this idea. For example, can you maybe collect CREBBP and KRAS mutant patients from online database(like TCGA), and to see whether this mutation predicts bad prognosis?

***Response:***

We acknowledge the importance of further validating these findings. We propose to access publicly available databases, such as The Cancer Genome Atlas (TCGA), to collect data on patients with confirmed CREBBP and KRAS mutations. Through this analysis, we aim to investigate whether the presence of these mutations correlates with poor prognosis in iCCA. Specifically, we plan to perform survival analyses to assess overall survival and disease-free survival rates in relation to these genetic alterations. By employing appropriate statistical methods, we will validate any observed associations and explore the underlying mechanisms that may contribute to this relationship. This additional research will not only enhance the robustness of our initial findings but also provide valuable insights into the clinical significance of CREBBP and KRAS mutations in the management of iCCA patients.
6.	As described in this article, CREBBP mutation might lead to dysregulated epigenetic alterations, therefore causing silence of some tumor suppressing genes. Could you maybe identify potential CREBBP affected tumor suppressing genes and test the expression levels with sample at hands to further confirm this idea? Despite the limitations listed above, this study is still providing us useful information on genetic landscape of iCCA among Thai population, making it a critical reference for further biomarker identification.

***RESPONSE:***

To address this, we accessed the TCGA Cholangiocarcinoma (CHOL) dataset via cBioPortal to evaluate the co-occurrence of *CREBBP* and *KRAS* alterations and their impact on patient survival. We have added a paragraph detailing this database analysis and its implications regarding endemic vs. non-endemic iCCA to the **Discussion**.

“Interestingly, our *in-silico* analysis of the TCGA-CHOL cohort revealed that the *CREBBP* and *KRAS* co-mutation is relatively rare in that dataset, preventing robust survival analysis. However, this is a highly expected and biologically significant finding: the TCGA cohort is predominantly composed of Western, non-fluke-associated iCCA patients, whereas our cohort consists of Thai patients with OV-endemic iCCA. The unique enrichment of this *CREBBP/KRAS* signature in our dataset strongly suggests it may be a distinct molecular hallmark of fluke-associated cholangiocarcinogenesis.”

**Reviewer #3:**

The authors use a targeted mutation panel to investigate the determinants of intra-hepatic cholangiocarcinoma in the presence of infection. The work represents a novel contribution to the field since the reported associations between CREBBP and KRAS genetics in Thai populations, which have a disproportionately large incidence rate of the disease, have not been previously reported on (although mutations in KRAS have been identified as a driver gene in Chinese - https://www.nature.com/articles/ncomms6696). The authors stress the difficulties of obtaining sufficient numbers of subjects to participate in the study resulting in small sample sizes for case-control and case-case comparisons. The overall conclusion is that 13 variants in two genes describe the mutational landscape of the disease / normal population. However, given the aforementioned small sample sizes, and demographic inequalities between test subjects assigned to study groups, I am unconvinced that these data support the conclusions. In particular, their may exist many other mutations on a genome wide scale that stratify these groups in a similar, if not better manner. Considering this fact, the authors might consider ammending their grand title to more closely reflect the findings from the study. Given the sample size issues, I am not convinced the statistical tests performed here are sufficient to test the hypothesis, that the variant frequencies significantly differ between groups. For example, in the demographics section, and text, the bias in age and sexes between test subjects in the study groups is emphasized, however, statistical methods that allow modelling and testing under these conditions have not been adopted (see multivariate logistic regression, Mantel-Haenszel tests , Stratified Fisher's Exact Test or conditional logistic regression as alternatives). Since the control population is limited to females only, it would seem only appropriate to report the trend in females, or to acquire approximated estimates of these frequencies in males from one of the South East Asian BioBanks.

Given the importance of studying these mutation profiles in under-represented or difficult to obtain population cohorts, I'd consider accepting the paper after making some MAJOR changes. First, some attempt to compare / contextualise the results/mutation frequencies among other South East Asian populations (chinese/japanese for example) and published studies on iCC. I'd also like the statistical tests to be re-evaluated considering age and where possible, sex as factors, and a power calculation to exemplify why these studies can only be used as guidelines or as hypothesis generators rather than considered hard evidence. [ The raw frequency differences are large, hence I anticipate the existing trends would be preserved under different test methodologies.] The methods section simply states frequencies were generated as input to chi-square or Fishers Exact test. Again, given the sample sizes, chi-square test results would seem inappropriate in this case.

The results and discussion sections should then include information around the biology eluded to from the mutation profiling results, and the observed population differences. For example., are the rates of OV infection similar across the asian populations ... and are the particular KRAS mutations preserved. Can the results be used to make a population specific signature ?
I feel with a few additions, the work would be significantly elevated to accepted status.

***RESPONSE:***

We agree with the reviewer that our original title was too broad and inadvertently implied a genome-wide discovery rather than a targeted panel approach. To accurately reflect our methodology and avoid overstating our findings, we have revised the title of the manuscript.

- Revised Title: “*Targeted Mutational Profiling of KRAS and CREBBP in a Thai Population with Opisthorchis viverrini-Associated Intrahepatic Cholangiocarcinoma*”
- Statistical analysis: We have completely overhauled our statistical analysis in accordance with the reviewer's expert recommendations:
  - We have replaced all Chi-square tests with Fisher’s Exact tests to properly handle small cell counts.
  - To account for the confounding effects of age and sex, we have applied multivariable logistic regression models to evaluate the independent association of the variant frequencies between groups.
  - Because our control group consists solely of females, we have conducted a stratified analysis specifically looking at the mutational trends within the female-only cohort to eliminate sex-bias in case-control comparisons.

These revised statistics have been updated throughout the **Methods**, **Results**, and **Tables**.

- We have performed a retrospective power calculation (added to the **Methods** section) which confirms that due to the limited sample size, our study has restricted statistical power for detecting rare variants. Consequently, we have substantially revised our **Discussion** and **Conclusions** to temper our claims. We now explicitly state that our findings should not be interpreted as definitive "hard evidence," but rather as a highly suggestive "hypothesis-generating" pilot study. This limitation is prominently featured to guide future, large-scale, multi-center studies in Southeast Asia.

"*Post-hoc* power analysis indicates that our small sample size limits the statistical power to detect low-frequency variants. Therefore, this study is designed as a hypothesis-generating investigation, and our identified mutation profiles serve as guidelines requiring validation in larger, well-powered epidemiological cohorts."

- We thank the reviewer for prompting this crucial discussion and for providing the excellent reference regarding *KRAS* in Chinese populations (*Zou et al., Nature Communications, 2014*). We have added a major new section to our **Discussion** to contextualize our findings:
  - We emphasize that OV is highly endemic specifically to the Greater Mekong Subregion (Thailand, Laos), whereas iCCA in China and Japan is more frequently driven by HBV/HCV infections or *Clonorchis sinensis*.
  - We reference the provided *Nature Communications* study to note that while *KRAS* is a recognized driver gene in Chinese iCCA cohorts, the specific combination and high frequency of the *CREBBP/KRAS* alterations observed in our Thai cohort appear unique.
  - We hypothesize that this distinct mutational landscape is likely a direct result of the specific, localized inflammatory mechanisms of chronic OV infection. Therefore, we propose that the *CREBBP/KRAS* profile may indeed represent a Thai/OV population-specific signature for iCCA, rather than a pan-Asian feature.

"In contrast to our findings in the Thai population, previous comprehensive genomic profiling in Chinese cohorts has identified different dominant driver pathways, though KRAS remains a significant driver (Zou et al., Nat Commun, 2014). This divergence likely stems from different etiological backgrounds, as Chinese and Japanese iCCA cases are predominantly associated with viral hepatitis or *Clonorchis sinensis,* whereas our cohort is strictly exposed to endemic OV. Consequently, the CREBBP/KRAS mutational profile observed here may represent a unique, population-specific signature for OV-endemic iCCA."
